# The More-or-Less Morphing Face Illusion Revisited: Perceiving Natural Transient Changes in Faces Despite Fast Saccades

**DOI:** 10.1177/2041669520943218

**Published:** 2020-07-23

**Authors:** Sandra Utz, Claus-Christian Carbon

**Affiliations:** Department of General Psychology and Methodology, University of Bamberg; Department of General Psychology and Methodology, University of Bamberg; Bamberg Graduate School of Affective and Cognitive Sciences, University of Bamberg

**Keywords:** face perception, face processing, more-or-less morphing face illusion, saccades, visual suppression, emotional expression, eye movements, fixation

## Abstract

van Lier and Koning introduced the *more-or-less morphing face illusion*: The detection of changes in a constantly morphing face-sequence is strongly suppressed by fast eye saccades triggered by a moving fixation dot. Modulators of this intriguing effect were investigated with systematically varied facial stimuli (e.g., human faces from varying morphological groups, emotional states) and fixation location. Results replicated the overall pattern of moving fixations substantially reducing the sensitivity to detect transitions. Importantly, a deviation from real to perceived changes could only be detected when faces were altered in a way not happening in real world—by changing identity. When emotional states of faces were changed, people were capable of perceiving these changes: A situation very similar to everyday life where we might quickly inspect a face by executing fast eye saccades but where we are still aware of transient changes of the emotional state of the very same person.

## Introduction

**Figure 1.**
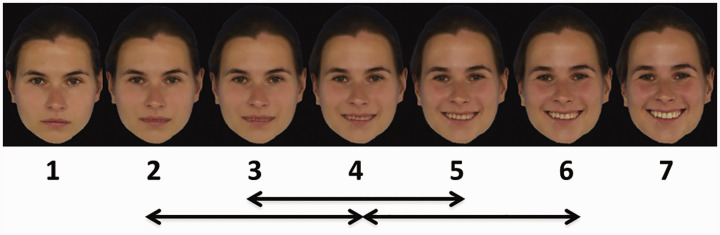
Example for a (Emotional Expression) Morphing Sequence; Real Morphing Range Indicated by Arrows (Following the Idea of van Lier & Koning, 2014).

**Figure 2.**
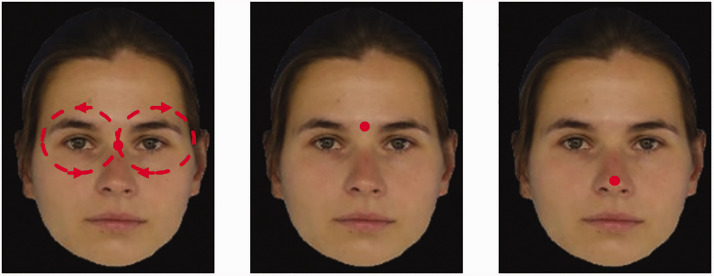
Example for the Three Different Types of Fixation. Left: clockwise moving dot (*moving*) following the moving dot procedure in van Lier & Koning (2014), middle: stationary dot between eyes (*stationary eyes*) following the static dot procedure in van Lier & Koning (2014); right: stationary on tip of the nose (*stationary nose*)—this condition was only realized for the *original* condition.

van Lier and Koning (2014) provided with their *more-or-less morphing face illusion* yet more evidence that eye movements strongly impact our perception, here particularly in terms of sensitivity of detecting ongoing changes in the background of a scene, that is, the nonfocused visual information. The authors showed quite impressively that as soon as beholders start fast eye saccades by following a to-be-fixated moving dot, the sensitivity to perceive changes within a sequence of animated transitions between two faces in the nonfocused area of a scene was substantially reduced. For their original experiment, they utilized artificially generated (FaceGen modeller©) basic faces (Face 1 to Face 7) which differed in their facial properties (e.g., position of eye brows). Based on these faces, transition sequences were generated by morphing faces, actually between Face 2 and 4, 3 and 5, and 4 and 6, respectively, that is, the real face morphing range consisted always of only two faces (see an example in Figure 1). Each sequence composed of 50 frames, going from one to the other face and vice versa. During watching a sequence, participants had to fixate either on a stationary red dot situated right between the eyes or on the same red dot, but this time moving in a figure-of-eight trajectory (see Figure 2). After each sequence, participants had to choose the two faces they thought the sequence was made of (start and end face of sequence). If they chose, for instance, Face 2 and Face 6 as the start and end face of the sequence, their selected morphing range (Δfaces) was 4. Results from this study showed that with a moving fixation dot, changes were perceived significantly smaller—or hardly noticed at all (*M* = 1.19)—than with a stationary fixation dot between the eyes (*M* = 2.85). The main mechanism which was suggested to underlie the effect is a reduced sensitivity for transient signals during executing (fast) eye movements (e.g., van Lier & Koning, 2014). Due to this reduced sensitivity, they will underestimate the degree of changes. Interestingly, van Lier and Koning (2014) further revealed an overestimation of the real morphing range when they had to fixate on a stationary fixation dot—they assumed that this overestimation happened as a result of a figural face aftereffect (Carbon & Ditye, 2011, 2012; Tangen et al., 2011; Webster & MacLin, 1999): through prolonged stationary fixation (between the eyes), differences in faces are perceived larger than they actually are. 

### The Present Study

Motivated by the preliminary explanation van Lier and Koning (2014) offered in their initial contribution, first of all, we aimed at replicating the study in order to test the robustness of the original effect which was documented before eight participants only. Furthermore, we wanted to further qualify the effect by differentiating the material between transitions which typically happen in real life (here: emotions) and which do not happen in real life (identity). In the original study, this demarcation line was not drawn as the employed changes of the position of eyebrows, for instance, can be interpreted as expressive as well as identificatory elements. We further varied our stimuli addressing identity aspects by different levels of *face expertise* (Schwaninger et al., 2003). Therefore, we employed faces of high expertise, so-called same-race faces stemming from the same morphological group of the participants (White faces), as well as faces of lower expertise: faces from a different morphological group (Black faces). In addition to faces from another species, here monkeys, were employed, reflecting very low expertise of processing faces. Rationale for using these different levels of expertise was that with increasing expertise people are more effective in processing such faces in terms of perception (MacLin & Malpass, 2003) and memory (Malpass & Kravitz, 1969; Pascalis & Bachevalier, 1997)—so if the effect between moving and stationary fixation is based on expertise, decreasing effects with increasing expertise should be found. To ensure that the original effect was not an artifact due to artificially generated faces, photographic depictions of real faces instead of computer generated ones were used. Finally, to test van Lier and Koning’s (2014) hypothesis about figural face aftereffects being responsible for the overestimation of perceived changes in the condition with a stationary fixation, an extra condition was added where the stationary fixation dot between the original position (between the eyes) was placed to a new position (stationary on the tip of the nose). For social interactions, the typical area in faces is located very closely to the eyes area (between eyes). Therefore, fixating on this area could lead to specific expertise-based processes connected to characteristic adaptation or figural face aftereffects. To rule that mechanism out and to include this as an additional variable, we decided to add an additional fixation area, where we cannot rely on expertise-based, optimized processes, because we do not typically fixate at the nose area and do not interact with this area.

## Method

### Participants

Thirty-one participants were tested with ages ranging from 18 to 28 years. All participants had normal or corrected-to-normal vision (assessed by a standard Snellen eye chart test) and all except one normal color vision (assessed by a short version of Ishihara color test)—the participant with nonnormal color vision was excluded from further analyses. All the remaining 30 participants (*M*_age_ = 20.7 years; *SD*_age_ = 2.3; six males) were Caucasian—all of them being undergraduates of the University of Bamberg. They received course credit points for their participation. They had no prior experience with the present task and were naive to the purpose of this experiment. The study was conducted according to the principles expressed in the Declaration of Helsinki and according to ethical principles of the German Psychological Society (Deutsche Forschungsgemeinschaft,) and the Association of German Professional Psychologists (Bundesverband Deutscher Psycholog*innen). Each participant was made aware of their right to withdraw themselves and their data from the study without consequences and without giving reasons. Written informed consent was given by each participant. The details and the rationale of the study were discussed with each participant on completion of the experiment.

### Apparatus

Participants were seated approximately 55 cm in front of a 58.4 cm. Samsung Syncmaster 2233 TFT monitor running at a 1,680 × 1,050 pixel screen resolution with a refresh rate of 60 Hz controlled by a Thermaltake LanboxLite PC. Participants responded by pushing keyboard buttons between 1 (= *first possible picture of the sequence*) and 7 (= *last possible picture of the sequence*) to indicate the starting and end picture of the perceived sequence. To ensure that the participants were not moving their heads, a chin rest was adjusted at an individually adjusted, comfortable height (50 cm away from the plane of the monitor). To ensure participants followed the instructions and fixated on the red dot, eye movements were monitored during the experiment using the EyeLink 1000 © system (max. sample rate of 2,000 Hz, actually running at 1,000 Hz; spatial accuracy of 0.25°‒0.5°, manufactured by SR Research Ltd. ©, Canada). While the viewing was binocular, the recording was monocular (right eye), following the standard protocol of such studies (e.g., Lykins et al., 2006).

Stimuli, trials, and experimental blocks were created with the up-to-date version of WinPython (version 2.7.9) ensuring high precision in executing the correct timing of the study.

### Stimuli and Materials

A questionnaire gathered the Caucasian participants’ contact to Black people in their daily life (adapted, based on a translated version of the original questionnaire by Hancock & Rhodes, 2008).

Morphing sequences each consisting of two photos of different faces (taken from BA-DADA face database, © by CCC at University of Bamberg; a set of face images, provided to CCC by Professor Colin Tredoux, University of Cape Town, South Africa; monkeys from Mollison & Goodall, 2006) were created with Abrosoft FantaMorph Deluxe 5, Version 5.4.6 (Copyright © 2002-2015 Abrosoft Co), that is, the starting face turns into the end face and back and forth. Areas in which facial depictions were presented were approximately 17 cm high and 12 cm wide. There were two different sequences with *Black* faces (one female and one male), two with *White* (*Caucasian*) faces (one female, one male), and two with *monkey* faces (one female and one male). All of these three conditions reflected nonnatural transitions—in real life we cannot observe such identificatory changes within one face. We also employed one condition where we used transitions of different *emotional* facial expressions (one female, one male) which do reflect typical natural transitions in real life (changing from neutral to happy and from happy to sad). Finally, we generated two sequences with White faces (one male and one female) where a mixture of changes was employed (eyebrows moved upward, mouth, and eyes became taller), similarly to the stimulus used by van Lier and Koning (2014). This last condition will be called *original* in the following (note, however, that we used *real* faces instead of the computer generated ones used by van Lier & Koning (2014) which appear quite artificially).

Morphing sequences were always created between Faces 2 and 4, 3 and 5, and 4 and 6 of each series (each composed of 50 frames, going from one to the other face and vice versa), that is, the real face morphing range consisted always of only two faces (see Figure 1 for an example stimuli sequence).

During an approximately 50-second presentation time, each sequence was run 8 times (i.e., each cycle lasted approximately 6 seconds). Each of the five conditions (*Black*, *White*, *monkey*, *emotional* faces, and the *original* morphing sequence replicated from van Lier & Koning, 2014) was realized by two different versions plus two fixation types: (a) with a red dot either moving in a figure-of-eight trajectory^1^ and (b) with a stationary fixation dot between the eyes. For the *original* version, we added the *stationary nose* condition with a fixation dot on the tip of the nose. Figure 2 depicts examples for all three types of fixation. In total, there were 62 different sequences: 5 conditions × 2 versions × 3 different start/end points (Faces 2 to 4, 3 to 5, 4 to 6) × 2 types of fixation + 2 versions of the sequence with fixation dot on tip of nose (*nose stationary*). Each participant was presented with a selection of 20 sequences (equal frequency of all different conditions) plus the two versions of the nose stationary condition (one male, one female; either Faces 2 to 4, 3 to 5, 4 to 6), that is, in total 22 sequences.

### Procedure

**Figure 3.**
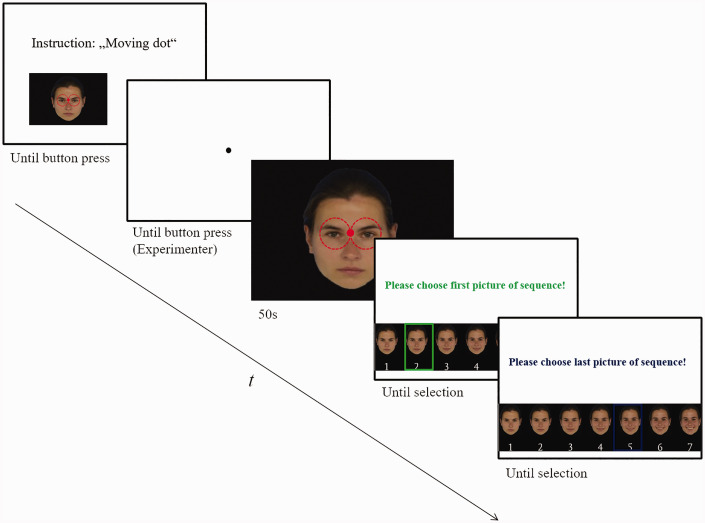
Example for One Trial With Moving Fixation. During the approximately 50 seconds morphing sequence participants had to fixate on a moving dot. Afterwards, they had to choose the start and end picture of the presented sequence.

After completion of the questionnaire regarding their contact to Black people in their daily life, the experiment started. Participants were introduced to the different types of fixation and were instructed to fixate at any time. As the type of fixation could change from trial to trial, a specific instruction was presented before each sequence, that is, whether in the following sequence, the red fixation dot would move in a figure-of-eight trajectory or would be stationary either between the eyes or on the tip of the nose. Participants started the sequences by pressing a key on the keyboard. After watching the sequence, they had to choose their presumed start of the sequence, that is, the face the sequence started with, and thereafter the ending, that is, the face the sequences ended with (and started to return to the starting one) out of seven optional pictures (see Figure 1 or Figure 3). After their selection, the instruction for the next sequence appeared. An example for the time course of a trial can be retrieved from Figure 3.

## Results

Morphing ranges assessed by participants (perceived ranges) were compared to the real morphing range of two faces and compared among the different types of stimulus and fixation conditions.

**Figure 4.**
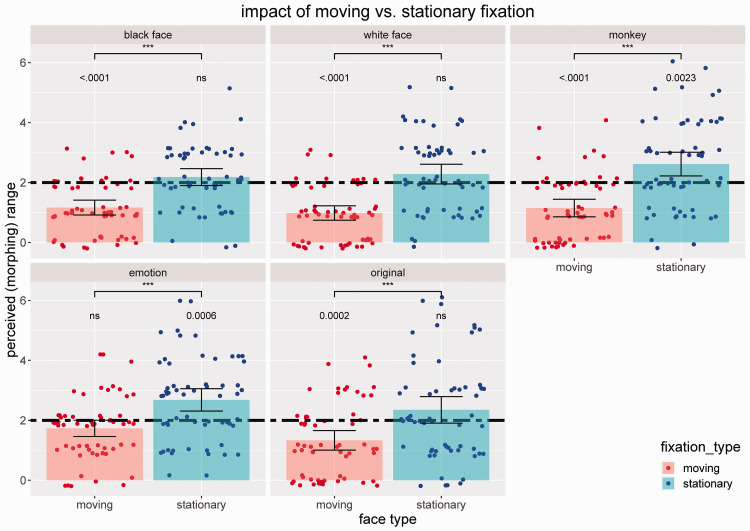
Perceived Morphing Range With a Moving Fixation Dot (red) Versus a Stationary Fixation Dot Between the Eyes (Blue), Split by All Stimulus Types. Bars show mean values, and dots show individual data. The real range (=2.0) is marked by the two-dashed horizontal line. *p*-values are based on non-parametric Wilcoxon tests with asterisks indicating significant differences between moving and stationary fixation types (***p < .001; one-sided) and exact *p* values indicating significant differences between respective condition and real morphing range (two-sided). Error bars represent 95% confidence intervals.

The initial graphical inspection of the data (Figure 4) shows the clear effect of much lower perceived morphing ranges when participants had to fixate on a moving fixation dot (as compared to a stationary dot), which replicates the general finding of van Lier and Koning (2014). This observation was validated by further statistical testing (see also Figure 4) revealing medium to large effects for the stimulus types original (Cohen’s *d* = 0.703) and emotion (*d* = 0.783) and large effects for Black faces (*d* = 1.027), monkey faces (*d* = 1.137), and White faces (*d* = 1.215) according to Cohen (1988). The overestimation of perceived ranges was much less pronounced compared to the data of the original paper; actually, we could only observe a significant difference from 2.0 (the real range) for the monkey face and emotion stimulus type. For sensitivity of perceiving changes in the face sequences when fixating on a moving dot, we revealed significant decreases for all but the emotion condition. Analyzing the corresponding effect sizes, we obtained the largest effect sizes for White faces (*d* = 1.611; very large effect), followed by Black faces (*d* = 1.276; large effect) and monkey faces (*d* = 1.106; large effect)—for the original condition, we gained a medium effect (*d* = 0.775). The selective effect of the decrease of sensitivity for conditions where the sequences contain transitions between identities, which we cannot find in real life (a face at one specific position does not change the identity from one moment to another), indicates a potentially very important phenomenon: Although a moving fixation dot that triggers fast saccades significantly decreases the sensitivity of perceiving changes in the nonfocused area of a scene, we seem to still register such changes when they stem from natural transitions which we typically experience in everyday life.

**Figure 5.**
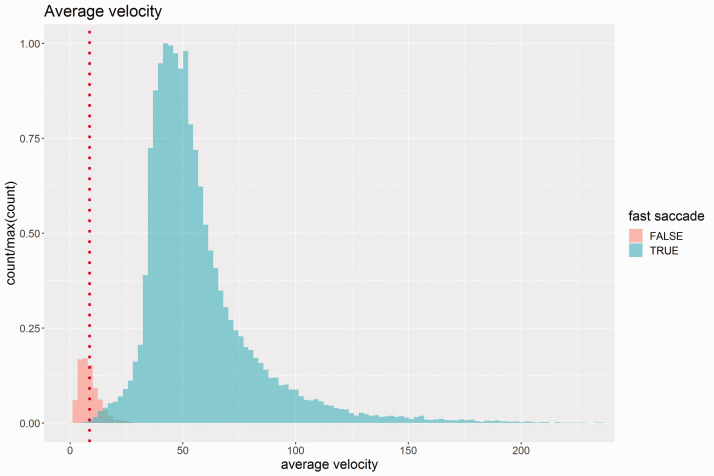
Histogram of Average Velocities of the Eye Saccades for Moving Dot Condition Across all Employed Stimuli. Red vertical line shows the velocity of the moving point.

To test whether participants actually showed fast saccades, we calculated the average velocities for the moving dot condition. The histogram of average velocities in Figure 5 shows a clear and typical bimodal distribution (see Komogortsev & Karpov, 2013). One subdistribution of eye movements showing average velocities around the actual speed of the moving dot (17.2 deg/s) with relatively low number of cases and another subdistribution with much faster eye movements. The first subdistribution is compatible with smooth pursuit, the latter with fast saccades. Overall, about 93.4% of all eye movements for the moving dot condition were classified as fast saccades (average velocities greater than 30 deg/s) which shows that this experimental conditions effectively triggered this type of eye movement which is known to suppress visual inputs (Krekelberg, 2010).

**Figure 6.**
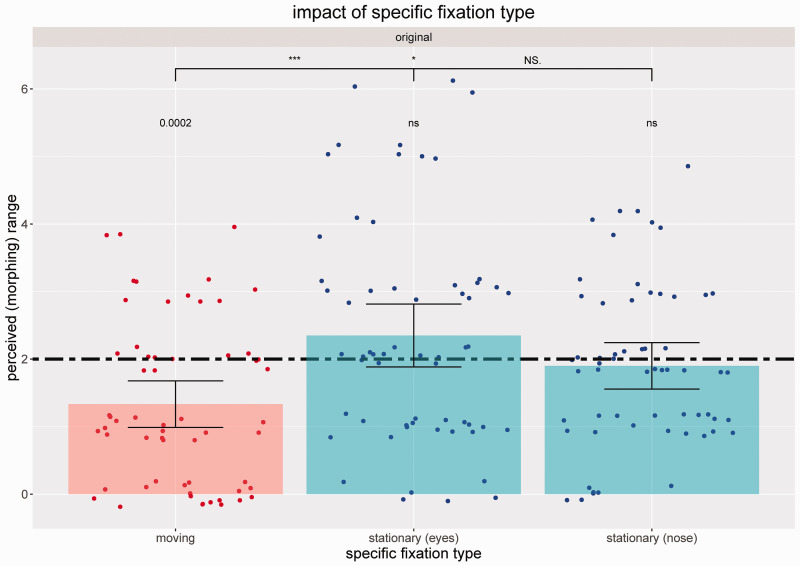
Perceived Morphing Ranges (Bars: Mean Values, Dots: Individual Data) for the Specific Fixation Types: Moving Dot, Stationary Dot Between Eyes and Stationary Dot on Tip of Nose (Real Range of 2.0 Is Marked by a Two-Dashed Horizontal Line). *p* Values are based on non-parametric Wilcoxon tests with asterisks indicating significant differences between specific fixation type conditions (*p < .05, ***p < .001; two-sided) and exact *p* values indicating significant differences between respective condition and real morphing range (two-sided). Error bars represent 95% confidence intervals.

The analysis of specific fixation types (moving dot vs. stationary dot between the eyes vs. stationary dot on the tip of the nose; all for the *original* stimulus condition only) showed a small numerical yet nonsignificant difference of the perceived morphing range for the stationary eyes versus stationary nose condition (Figure 6) but a significant difference between the moving dot and the stationary eyes condition (*d* = 0.703; medium effect) as well as between the moving dot and the stationary nose condition (*d* = 0.465; small effect). This underlines once more the robustness of the effect, at least for non-naturally changing stimuli (here utilized via the *original* stimulus type) which might not be expected by the viewer.

## Discussion

First of all, this study was able to closely replicate results by van Lier and Koning (2014) illustrating the strong replication power of basic perceptual phenomena very nicely. We could also expand the external validity by successfully transferring the phenomenon to more realistic, that is, actual human faces: Again, we were able to show a drastic decrease in sensitivity of perceiving changes in faces during fixating a moving dot in a figure-of-eight trajectory superimposed on a morphing face sequence. If this effect is based on expertise, we expected decreasing effects with an increasing expertise. Data, however, showed no difference for different levels of expertise and therefore arguing against an influence of expertise on the perception of the illusion. A deviation from real to perceived changes could, however, only be detected when faces were altered in a way which is not happening in real world—by changing the identity. The overestimation of changes in faces when fixating on a stationary dot could not be fully replicated as we failed to show this effect for most of our employed stimulus types. We could only find this effect for the monkey faces and emotional face condition. Whether the original finding in this respect was due to a larger sampling error because of a very small sample size (*N* = 8!) or was based on different criteria or sensitivity aspects or just by the specific employment of artificial faces (i.e., faces used in this study were more realistic and therefore more familiar), we do not know. For the specific paradigm used and for both stationary fixation locations employed, we obtained a perceived morphing range which was quite similar to the real morphing range of 2. Thus, we are not sure whether van Lier and Koning’s (2014) idea to propose a figural aftereffect being responsible for the overestimating behavior with a stationary dot is realistic as typical figural aftereffects unfold very fast and quite powerful and sustainable (Carbon & Ditye, 2011; Mueller et al., 2020).

The *more-or-less morphing face illusion* by van Lier and Koning (2014) has demonstrated how capable human-made illusions are to understand human perception (Carbon, 2014). When following a moving dot with fast saccades, this has a dramatic decrease in the sensitivity to detect visual changes going on in the nonfocused area of a scene. Most importantly, this visual suppression (Krekelberg, 2010) of the signal during fast saccades seems not to be fully rigid but relies on the quality of the visual field. When we perceive a face in the nonfocused area of a scene, there are potentially a lot of dynamics going on which will alter the visual field, for instance, movements of the body, head, and also within the face finer movements showing specific eye blinks, eye movements, and facial expressions. Whereas some of this information might not be very informative and so could be dismissed or easily extrapolated from a given situation, others seem to be highly relevant for a social situation. One group of special candidates for such relevant information are facial expressions, especially those expressing emotional states. Actually, within the clear limits of this study, exactly these kinds of information were still accurately detected by the participants despite following the moving dot, because this represents a situation very similar to everyday life where we might quickly inspect the face of our counterpart by executing fast eye saccades but where we are still aware of transient changes of the emotional state of the very same person. However, neither a change of identity nor any other non-transient quality of that person would be expected. Therefore, on basis of the results of this study, important further conclusions can be drawn about possible underlying mechanisms and the specific impact of individual variables on the whole perceptual process of perceiving changes in faces.

## Note

1 The demo by van Lier and Koning for the illusion of the year contest 2011 contained a simple circle. However, we are referring to the moving fixation used in the van Lier and Koning (2014) journal paper instead.
